# A New Peracetylated Oleuropein Derivative Ameliorates Joint Inflammation and Destruction in a Murine Collagen-Induced Arthritis Model via Activation of the Nrf-2/Ho-1 Antioxidant Pathway and Suppression of MAPKs and NF-κB Activation

**DOI:** 10.3390/nu13020311

**Published:** 2021-01-22

**Authors:** María Luisa Castejón, Catalina Alarcón-de-la-Lastra, María Ángeles Rosillo, Tatiana Montoya, Jose G. Fernández-Bolaños, Alejandro González-Benjumea, Marina Sánchez-Hidalgo

**Affiliations:** 1 Department of Pharmacology, School of Pharmacy, University of Seville, 41012 Seville, Spain; mcastejon1@us.es (M.L.C.); calarcon@us.es (C.A.-d.-l.-L.); rosillo@us.es (M.Á.R.); tmontoya@us.es (T.M.); 2 Department of Organic Chemistry, School of Chemistry, University of Seville, 41012 Seville, Spain; bolanos@us.es (J.G.F.-B.); a.g.benjumea@irnas.csic.es (A.G.-B.)

**Keywords:** arthritis, CIA, inflammation, MAPK, NF-κB, Nrf2/HO1, oleuropein

## Abstract

Oleuropein (OL), an olive tree secoiridoid and its peracetylated derivate (Per-OL) have exhibited several beneficial effects on LPS-stimulated macrophages and murine experimental systemic lupus erythematosus (SLE). This study was designed to evaluate dietary Per-OL in comparison with OL supplementation effects on collagen-induced arthritis (CIA) murine model. Three-weeks-old DBA-1/J male mice were fed from weaning with a standard commercial diet or experimental enriched-diets in 0.05 % (*w*/*w*) OL, 0.05% and 0.025% Per-OL. After six weeks of pre-treatment, arthritis was induced by bovine collagen type II by tail base injection (day 0) and on day 21, mice received a booster injection. Mice were sacrificed 42 days after the first immunization. Both Per-OL and OL diets significantly prevented histological damage and arthritic score development, although no statistically significant differences were observed between both compounds. Also, serum collagen oligomeric matrix protein (COMP), metalloprotease (MMP)-3 and pro-inflammatory cytokines levels were ameliorated in paws from secoiridoids fed animals. Mitogen-activated protein kinases (MAPK)s and nuclear transcription factor-kappa-B (NF-κB) activations were drastically down-regulated whereas nuclear factor E2-related factor 2 (Nrf2) and heme-oxygenase-1 (HO-1) protein expressions were up-regulated in those mice fed with OL and Per-OL diets. We conclude that both Per-OL and its parent compound, OL, supplements might provide a basis for developing a new dietary strategy for the prevention of rheumatoid arthritis.

## 1. Introduction 

Rheumatoid arthritis (RA) is the most prevalent chronic, painful, and debilitating autoimmune disease in the world. It characterized by chronic synovitis leading to the progressive destruction of joints accompanied by systemic inflammation and the production of autoantibodies. Moreover, RA includes extraarticular manifestations, such as rheumatoid protuberances, pulmonary involvement or vasculitis and systemic comorbidities [1] affecting to the patient’s capacity to perform physical activities compared with the healthy population [2]. 

Although the pathogenesis of RA is multifactorial, substantial insights into RA pathophysiology suggest that various inflammatory pathways lead to an altered immune system and contribute to the joint damage during the disease. In particular, immune cells mainly B-cells, T-cells and macrophages and even fibroblast-like synoviocytes play pivotal roles in RA pathogenesis [3]. Autoreactive B-cells produce rheumatoid factors (RFs) and/or anti-citrullinated protein antibodies (ACPAs) and mediate T-cell activation through an expression of co-stimulatory molecules. According to the T-cells, they activate macrophages and fibroblasts and transform them into tissue-destructive cells producing a variety of pro-inflammatory chemokines and cytokines that perpetuate joint inflammation and aggravate tissue destruction at the chronic disease stage [4]. Nevertheless, several environmental and other factors such as multiple genetic, geography, socioeconomic status, diet/nutrients, alcohol, smoking, and host-microbiome also contribute to the risk of developing RA [5,6]. 

Understanding the mechanisms involved in RA pathogenesis has led in large part to the clinical development of therapeutic drugs targeting specific cells and molecules. In fact, the most successful therapeutic strategy in this disease include disease-modifying-anti-rheumatic drugs (DMARDs), such as methotrexate and cytokine-directed therapies including inhibitors of tumor necrosis factor (TNF) and interleukin (IL)-6 [7,8]. However, these drugs are effective only in a fraction of patients and have other limitations including high cost, the requirement of parenteral administration and important side effects. Thus, the search for novel treatments with a more benign safety profile is needed. In this sense, within the therapeutic approach of RA, nutritional therapy could be relevant because, in the last few years, it has been highlighted that determinate foods consumption has an influence on health outcomes [9].

The antioxidant and anti-inflammatory properties of biophenolic fraction from olive leaves and fruit from *Olea europaea L*., have been suggested as a potential application in several in vitro and in vivo models of inflammation studies [9,10]. Recently, we have reported the protective role of these compounds in other pathologies such as cancer, cardiovascular, neurodegenerative, ageing-related, and immunoinflammatory diseases [11]. It is well-known that extra-virgin olive oil (EVOO) contains significant amounts of monounsaturated fatty acid (MUFA) (oleic acid) and other minor but highly bioactive components including triterpenic alcohols, sterols, hydrocarbons (squalene), vitamins (α- and γ-tocopherols), β-carotene, phytosterols and numerous functional phenolic compounds [12]. Similarly, olive leaves which represent a waste product from olive harvest and pruning of olive trees, contain an even higher number of bioactive polyphenols than olive oil. Thus, it could be an useful font of natural compounds to study in different inflammatory conditions [13]. In particular, oleuropein (OL) the most abundant and characteristic polyphenolic compound in unprocessed olive leaves [14] is responsible for the major anti-inflammatory effects of olive leaf extract [15]. It has been well-established that OL possesses antioxidant and anti-inflammatory effects in lipopolysaccharide (LPS)-stimulated RAW 264.7 macrophages [16] inhibiting biosynthesis of pro-inflammatory cytokines, and regulating inflammatory response by inducible nitric oxide synthase (iNOS) and cyclooxigenase 2 (COX-2) suppression. Moreover, it has been described that OL down-regulated TNFα secretion in freshly isolated human peripheral blood mononuclear cells (PBMCs) culture [15] and the IL-1β-induced inflammation and oxidative stress in human synovial fibroblast cell line SW982 [17]. Moreover, OL has been shown protective effects on cartilage slowing down the progression of spontaneous osteoarthritis lesions in guinea pigs [18]. On the other hand, OL-aglycone is a product of hydrolysis obtained from OL by β-glucosidase on the parent glucoside. This compound was able to improve the clinical signs and improved histological status in the joint and paw from collagen-induced arthritis (CIA) mice decreasing the degree of oxidative and nitrosative damage [19]. However, presently, the anti-arthritic effects of OL on CIA have not been yet elucidated.

Moreover, the synthesis of OL derivatives with better pharmacokinetic and pharmacodynamic profiles than OL could be a strategy in the management of the inflammatory process. In fact, there are some studies which have shown the advantages of acetylated derivatives of natural compounds, since their lipophilic nature allows them to cross the cytoplasmic cell membranes and their uptake by cells, offering a possible protection of membrane components using a LPS-induced acute inflammation model in murine isolated macrophages [20]. Recently, we have reported that both OL and its new peracetylated derivative, peracetylated-OL (Per-OL), attenuated kidney injury in pristane-induced systemic lupus erythematosus (SLE) model inhibiting pro-inflammatory biomarkers overexpression via activation of heme-oxygenase (HO)-1/Nuclear factor E2-related factor 2 (Nrf-2) antioxidant pathway and suppression of the Janus kinase (JAK-STAT), nuclear transcription factor-kappa B (NF-κB) and mitogen-activated protein kinases (MAPK) activations [21]. 

Thus, taking this background into account, the present study was designed to evaluate, for the first time, the potential protective effects of dietary treatments of Per-OL derivative in comparison with its parent compound (OL). In addition to macroscopic and histological analyses, we determined the effects of these experimental diets on the production of inflammatory mediators and explored the signaling pathways involved. 

## 2. Materials and Methods

### 2.1. Reagents

OL was extracted from olive leaves according to reported literature [22], following the purification by column chromatography (CH_2_Cl_2_-MeOH 10:1→5:1). The synthesis of Per-OL was carried out from OL. These processes were carried out according to the methodology described by Castejon et al., 2019 [20]. 

### 2.2. Animals

To evaluate the beneficial effects of Per-OL and OL on CIA model, a total of 60 three-weeks-old male DBA-1/J mice (Janvier^®^, Le Genest St Isle, France) were maintained in our laboratory according to the conditions described by Rosillo et al., 2015 [23].

In particular, mice were randomized in five experimental groups (12 animals per group): (i) Naïve group (SD-Naïve); (ii) CIA Control group (SD-CIA), (iii) OL diet group enriched 0.05% (OL-CIA), (iv) Per-OL diet group enriched 0.05% (Per-OL 0.05-CIA) and (v) Per-OL diet group enriched 0.025% (Per-OL 0.025-CIA). 

### 2.3. Induction of CIA

RA was induced in 10-week-old male DBA-1/J mice according to the technique previously described by our research group [23,24]. Joint inflammation was scored visually in each paw, using a scale of 0–2 where 0 = uninflamed, 1 = mild, 1.5 = marked and 2 = severe. Scoring was performed by two independent observers without knowledge of the experimental groups. 

### 2.4. Histological and Immunohistochemical Analyses

After mice were sacrificed, knee joints were removed and fixed in 4% formaldehyde. After decalcification in 10% ethylenediaminetetraacetic acid (EDTA) specimens were processed for paraffin embedding. Tissue sections (7 µm) were stained with hematoxylin and eosin (H&E) to perform histological analysis. For immunohistochemistry assay of COX-2, the procedure was according to Rosillo et al., 2014 [25].

### 2.5. Study of Inflammatory Markers

Enzyme-linked immunoassay (ELISA) kits were used to measure serum levels of cartilage oligomeric matrix protein (COMP) (MD Biosciences^®^, Zürich, Switzerland) and matrix metalloproteinase-3 (MMP-3) (R&D Systems^®^, Abingdon, UK). Final paws were amputated above the ankle and homogenized in 1 mL of 10 mM 4-(2-hydroxyethyl)-1-piperazineethanesulfonic acid (HEPES) buffer, pH 7.4. Supernatants were used for determination of IL-17 (Peprotech^®^, London, UK), TNF-α, IL1-β (BD OptEIA^®^, San Jose, CA, USA), interferon (IFN)-γ and IL-6 (Diaclone^®^, Besacon Cedex, France). The results were measured at 450 nm using an ELISA microplate reader (BioTek^®^, Bad Friedrichshall, Germany). All measurements were carried out in duplicate.

### 2.6. Isolation of Cytoplasmic and Nuclear Proteins and Immunoblotting Detection

Frozen final paws were homogenized in liquid N_2_. Isolation of cytoplasmic and nuclear proteins was performed according to Rosillo et al., [23]. Protein concentration of the paw’s homogenate was measured following Bradford’s method [26]. Aliquots of supernatant that contain equal amount of protein (50 µg) were used to determine iNOS, p-p38, p-JNK, p-ERK, NF-κBp65, NF-κBp50, inhibitory of NF-κB (IκB)-α, Nrf2 (Cell Siganling^®^, MA, USA) and HO-1 (Enzo^®^, Madrid, Spain) proteins expression according by Western Blot [20]. The immunosignals were captured using Amersham Imager 600 (GE Healthcare^®^, Buckinghamshire, UK) and the signals were analyzed and quantified by an Image Processing and Analysis in Java (Image J^®^, Softonic, Barcelona, Spain). 

### 2.7. Statistical Evaluation

All values in the text and figures are expressed as arithmetic means ± standard error (S.E.M.). Data were evaluated with Graph Pad Prism^®^ Version 6.01 software (San Diego, CA, USA). The statistical significance of any difference in each parameter among the groups was evaluated by one-way analysis of variance using Tukey’s multiple comparisons test as post *hoc* test. *p* values of < 0.05 were considered statistically significant. In the experiments involving densitometry, the figures shown are representative of at least three different experiments performed on different days. 

## 3. Results

### 3.1. Dietary OL and Per-OL Treatments Alleviated CIA-Related Symptoms and RA-Induced Infiltration of Inflammatory Cells

**Figure 1:**
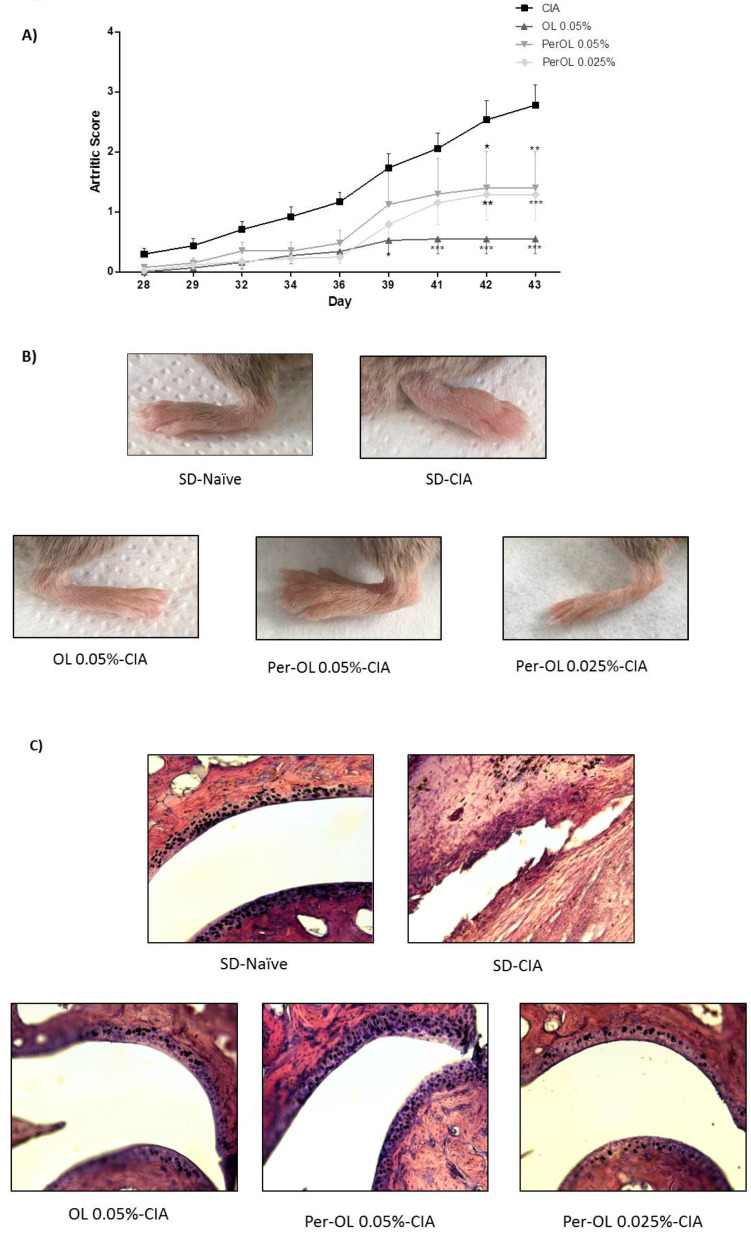
Time course of the arthritis macroscopic score (**A**), representative pictures (**B**) of hind paws at the end of the experiment (day 42). Histological analysis of the sections of knee joints stained with H&E on day 42 (**C**). Original magnification x20. SD-Naïve, non-arthritic mice feed with SD; SD-CIA, control arthritic group feed SD; OL 0.05%-CIA, arthritic group feed with enriched-diet with OL 0.05% *w*/*w* and Per-OL 0.05% and 0.025%, arthritic group feed with enriched-diet with Per-OL 0.05 and 0.025% *w*/*w,* respectively. Pictures show less damage at the cartilage. The images are representative of at least five experiments. Data represent mean ± S.E.M. *n* = 12. * *p* < 0.05; ** *p* < 0.01 and *** *p* < 0.001 vs. SD-CIA control group.

The development of RA was monitored until day 42. The time course of arthritic score (Figure 1A) shows that CIA control mice fed with normal standard diet (SD-CIA) presented a progressive development of clinical symptoms. On the contrary, the severity of RA symptoms was lower in those groups which were fed with OL 0.05% and Per-OL 0.05 and 0.025% experimental diets than in CIA control group from days 30 to 42. These results suggested that dietary OL and Per-OL could have a similar therapeutic behavior on going inflammatory arthritis. Figure 1B shows representative photographs of hind paws from the different experimental diet animal groups.

In addition, H&E staining revealed that histological features of the joint from sham animals were typical of normal structure with synovial membrane composed of synovial cells, collagen, and a clear synovial space. On the contrary, joints from SD-CIA mice exhibited histological changes indicative of severe arthritis, characterized by an extensive inflammatory cell infiltration into articular tissues, exudation into the synovial space, hyperplasia, and cartilage erosion. These histological features were less evident in those arthritic mice fed with OL and Per-OL experimental diets (Figure 1C). 

### 3.2. Effects of Dietary OL and Per-OL Treatments on Serum Pro-Inflammatory Biomarkers Levels in CIA Model

**Figure 2:**
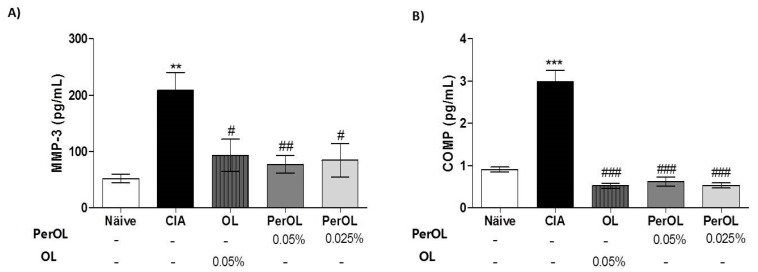
Measurement of serum levels of MMP-3 (**A**) and COMP (**B**) levels. They were measurement by ELISA commercial kits. Data represent mean ± S.E.M., *n* = 12. ** *p* < 0.01 and *** *p* < 0.001 vs. SD-Naïve control group, ^#^ *p* < 0.05, ^##^ *p* < 0.01 and ^###^ *p* < 0.001 vs. SD-CIA control group.

MMP-3 is well-known as a predictor for joint destruction in RA. We described an increment of circulating MMP-3 levels in SD-CIA control group mice in comparison to SD-Naïve control group (** *p* < 0.01 vs. SD-Naïve control group) whereas these levels were significantly reduced after Per-OL and OL dietary treatments at all doses assayed. It should be noted that the results obtained with the derived compound were even better in comparison with its parent compound (^#^ *p* < 0.05; ^##^ *p* < 0.01 vs. SD-CIA control group) (Figure 2A). Similarly, serum levels of the cartilage degradation marker, COMP, were significantly increased in SD-CIA mice when compared to SD-Naïve control group (*** *p* < 0.001 vs. SD-Naïve control group). However, Per-OL and OL-enriched diets significantly decreased serum COMP levels in CIA mice reaching similar levels to those observed in SD-Naïve control group (^###^ *p* < 0.001 vs. SD-CIA control group) (Figure 2B).

### 3.3. Dietary OL and Per-OL Treatments Suppressed the Production of Pro-Inflammatory Cytokines in CIA Model 

**Figure 3:**
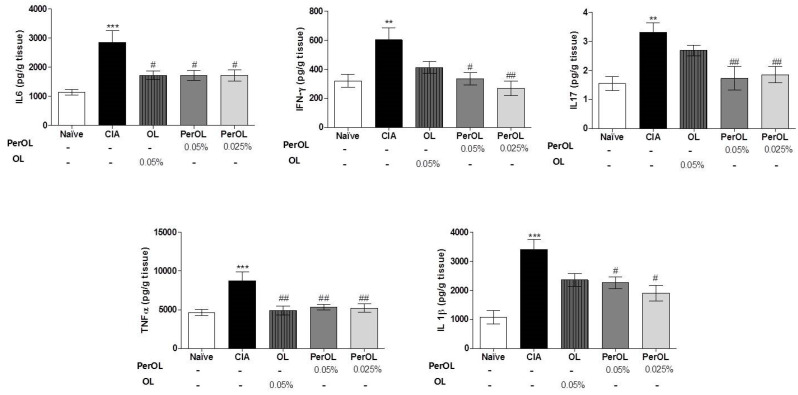
Measurement of pro-inflammatory cytokines: TNF-α, IL-1β, IL-6, IL-17 and IFN-γ levels in hind paw homogenates. These were determined by ELISA kits. Data represent mean ± S.E.M., *n* = 12. ** *p* < 0.01 and *** *p* < 0.001 vs. SD-Naïve control group, ^#^ *p* < 0.05 and ^##^ *p* < 0.01 vs. SD-CIA control group.

We studied if dietary Per-OL and OL treatments were capable of reducing the pro-inflammatory cytokines levels, which were involved in the pathogenesis of RA. We examined these pro-inflammatory cytokines levels in paws homogenates by ELISA. We could observe a higher increase on IL-1β, IL-6, IL-17, IFN-γ and TNF-α levels in paw homogenates from SD-CIA control group (** *p* < 0.01; *** *p* < 0.001 vs. SD-Naïve) when compared with SD-Naïve mice. Nevertheless, these levels were reduced significantly (^#^ *p* < 0.05 and ^##^ *p* < 0.01 vs. SD-CIA control group) on those mice were fed with 0.05% OL and 0.05% or 0.025% Per-OL experimental diets. It should be noted that the decrease on the cytokines IL-1β, IL-17 and IFN-γ observed after per-OL treatment was more evident when compared with its parent compound (Figure 3).

### 3.4. OL and Per-OL Experimental Diets Reduced COX-2 and iNOS Overexpressions

**Figure 4:**
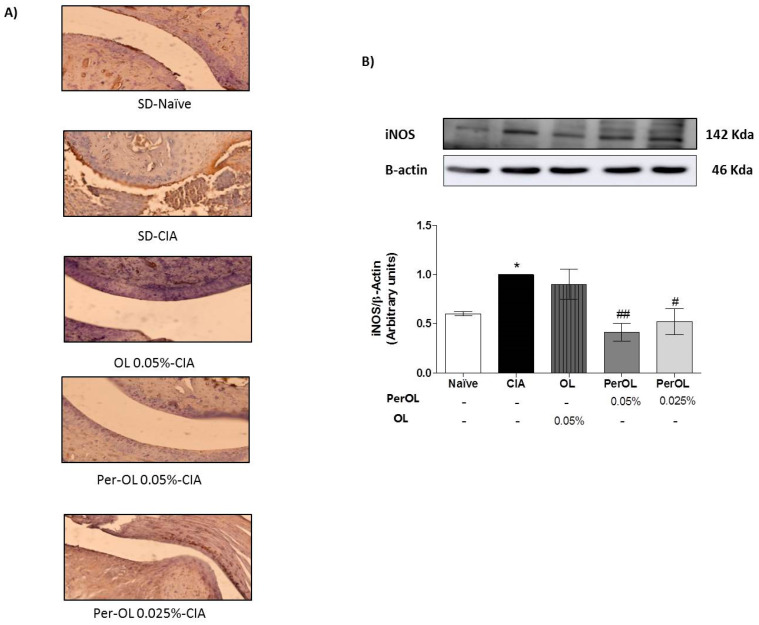
Expressions of COX-2 (**A**) and iNOS (**B**) in hind paws. iNOS protein expression was determined by Western Blot, quantified by densitometry analysis and normalized with respect to a house-keeping β-actin. COX-2 expression was determined by immunohistochemistry. Data represent mean ± S.E.M., *n* = 5. * *p* < 0.05 vs. SD-CIA control group; ^#^ *p* < 0.05 and ^##^ *p* < 0.01 vs. SD-CIA control group.

The effects of Per-OL and OL dietary treatments on CIA-induces COX-2 activation which was determined by immunohistochemistry on knee joint sections. We could observe a significant overexpression of COX-2 positive cells in SD-CIA control group, whereas Per-OL and OL experimental diets reduced notably the immunoreactivity for this pro-inflammatory enzyme (Figure 4A). In addition, the protein expression of iNOS was significantly up-regulated in paws homogenates from SD-CIA mice in comparison to SD-Naïve control group (* *p* < 0.05 vs. SD-Naïve control group) whereas, the protein expression of this pro-inflammatory enzyme was down-regulated in paws from arthritic mice fed with 0.05 % and 0.025 % Per-OL experimental diets (^#^ *p* < 0.05; ^##^ *p* < 0.01 vs. SD-CIA control group). It is interesting to emphasize that better results related to this inflammatory marker were obtained with the peracetylated compound (Figure 4B).

### 3.5. Effects of Dietary OL and Per-OL Treatments on MAPKs, NF-κB and Nrf2/HO Signaling Pathways

**Figure 5:**
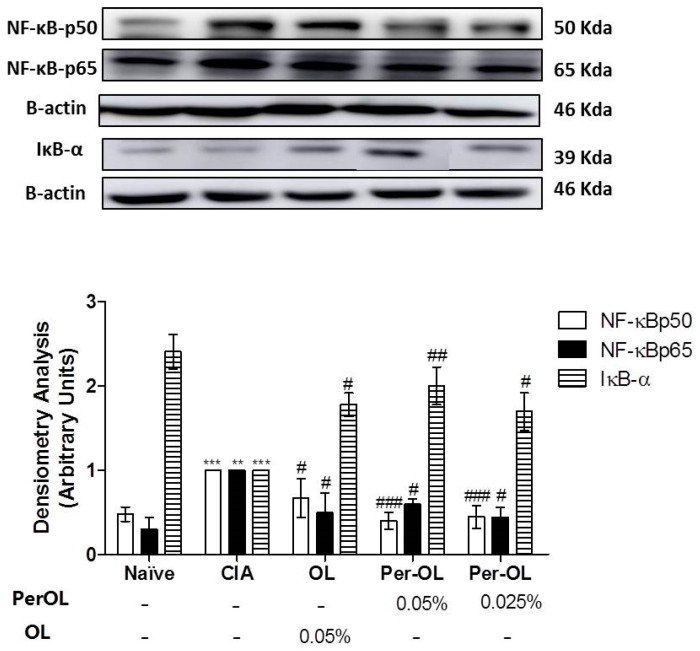
Protein expression of NF-κB-p65 and –p50 in nuclear fraction and IκB-α in cytoplasmic fraction of homogenates from hind paws. The expression was quantified by densitometry analysis and normalized with respect to a house-keeping β-actin. Data represent mean ± S.E.M., *n* = 5. ** *p* < 0.01 and *** *p* < 0.001 vs. SD-Naïve control group, ^#^ *p* < 0.05, ^##^ *p* < 0.01 and ^###^ *p* < 0.001 vs. SD-CIA control group.

We investigated the effects of OL and Per-OL experimental diets on NF-κB, MAPKs and Nrf2/HO-1 signaling pathways in paw homogenates. As shown in Figure 5, the expression of cytoplasmatic IκB-α was significantly reduced in SD-CIA control group when compared with SD-Naïve control group (*** *p* < 0.001 vs. SD-Naïve control group) which was accompanied by an overexpression of nuclear NF-κBp65 and NF-κBp50 subunits protein expressions in SD-CIA control group (** *p* < 0.01; *** *p* < 0.001 vs. SD-Naïve control group). On the contrary, dietary OL and Per-OL treatments prevented the IκB-α degradation (^#^ *p* < 0.05; ^##^ *p* < 0.01 vs. SD-CIA control group) and prevented the nuclear translocation of p50 and p65 subunits (^#^ *p* < 0.05; ^###^ *p* < 0.001 vs. SD-CIA control group). In fact, better results were also obtained from those animals fed with the peracetylated compound on NF-κBp50 and IκB-α expressions in comparison with OL.

**Figure 6:**
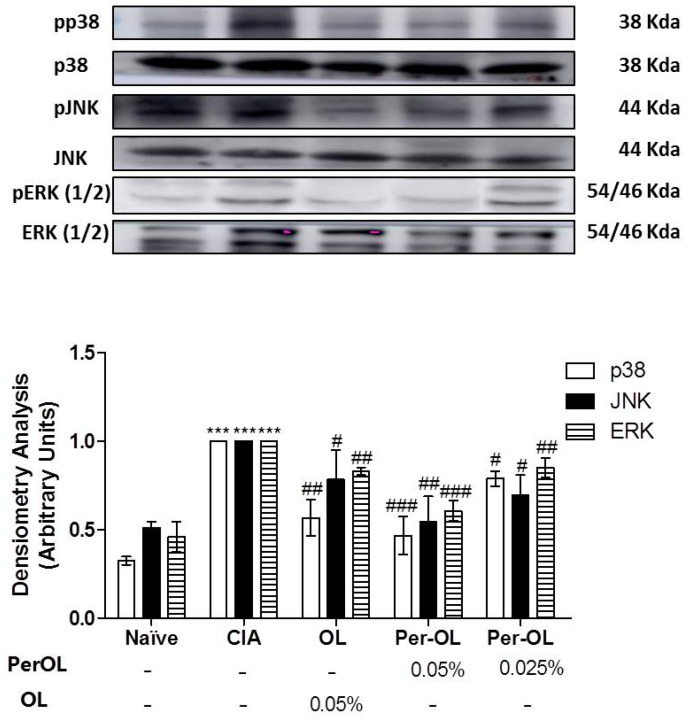
Effects of dietary OL and Per-OL on JNK, p38, and ERK_(1/2)_ MAPKs phosphorylation in hind paws homogenate. The expression of phosphorylated proteins was expressed related to the expression of the corresponding total protein. The expression was quantified by densitometry analysis and normalized with respect to a specific total protein. Data represent mean ± S.E.M., *n* = 5. *** *p* < 0.001 vs. SD-Naïve control group, ^#^ *p* < 0.05, ^##^ *p* < 0.01 and ^###^ *p* < 0.001 vs. SD-CIA control group.

MAPKs signaling pathways play a key role in the establishment of inflammation process. We investigated the effects of dietary Per-OL and OL treatments on MAPKs (extracellular signal-regulated kinases (ERK)_1/2_, c-Jun NH_2_-terminal kinase (JNK) and p38) signaling pathway activation in CIA mice. We observed a significant increase on ERK_1/2_, JNK and p38 phosphorylation in SD-CIA control group in comparison with SD-Naïve control group (*** *p* < 0.001 vs. SD-Naïve control group). Nevertheless, the phosphorylation degree of these MAPKs proteins was significantly ameliorated in those arthritic mice fed with all experimental diets assayed (^#^ *p* < 0.05; ^##^ *p* < 0.01; ^###^ *p* < 0.001 vs. SD-CIA control group) and more evident in those animals fed with Per-OL treatment in comparison with OL (Figure 6).

**Figure 7:**
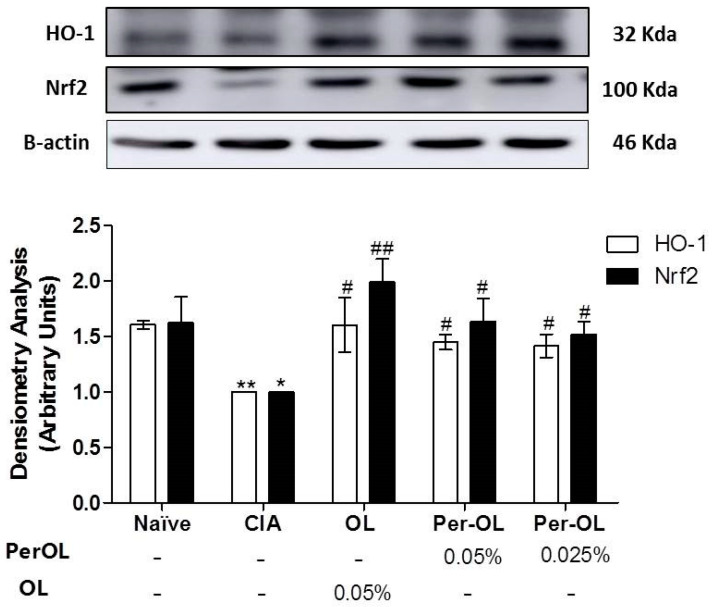
Effects of dietary OL and Per-OL treatment on HO-1 and Nrf2 up-regulation protein expressions in hind paws homogenates. The expression was quantified by densitometry analysis and normalized with respect to a house-keeping β-actin. Data represent mean ± S.E.M., *n* = 5. * *p* < 0.05 and ** *p* < 0.01 vs. SD-Naïve control group, ^#^ *p* < 0.05 and ^##^ *p* < 0.01 vs. SD-CIA control group.

Finally, the effects of dietary Per-OL and OL treatments on Nrf2 and HO-1 antioxidant pathway activation were also explored. We could observe a remarkable down-regulation of both Nrf2 and HO-1 protein expressions in SD-CIA mice (* *p* < 0.05; ** *p* < 0.01 vs. SD-Naïve control group) whereas Per-OL (0.05%) and OL (0.05%) experimental diets induced a significant Nrf2 and HO-1 overexpression in comparison with SD-CIA control group (^#^ *p* < 0.05; ^##^ < 0.01 vs. SD-CIA control group). In this study, the behavior of both compounds was comparable (Figure 7).

## 4. Discussion

RA is a chronic systemic inflammatory disorder affecting synovial lining of joints, bursae and tendon sheaths [27]. The objective of traditional pharmacological therapy of RA is to finish or reverse cartilage destruction and diminish the pain devoid of untoward effects. However, current treatments are not efficient in all patients and possess several disadvantages. Consequently, nutritional therapy as complementary and alternative medicine is under development as an innovative strategy in RA management [28]. In fact, it has been demonstrated that nutritive and non-nutritive dietary factors can affect the clinical outcome of rheumatic diseases. In this line, dietary polyphenols have been extensively investigated with regards to their antioxidant, anti-inflammatory, and immunomodulatory properties in many inflammatory chronic conditions, including RA. Several studies have shown that among bioactive polyphenols, hydroxytyrosol (HTy), hydroxytyrosol acetate (HTy-Ac), genistein, kaempferol and resveratrol seem to be effective in CIA, an experimental model for RA that shares several pathological, histological, and immunological features with human RA, by decreasing the production of pro-inflammatory cytokines, and activating the antioxidant defense system [25,29,30]. 

CIA induction resulted in the development of a pronounced synovitis associated with an autoimmune response against cartilage with inflammatory cells infiltrate and production of many cytokines and matrix-degrading, accompanying cartilage degradation and bone erosions [31,32]. Our results revealed, for the first time, that Per-OL and OL supplemented diets exhibited preventive effects in the development of inflammation and joint damage in SD-CIA. These results were correlated with an improved arthritis score and a reduction of inflammatory cells infiltration into articular tissue, synovial hyperplasia, and cartilage destruction. 

The secretion of MMPs, cytokines and growth factors contribute to the loss of normal homeostasis in the synovial joint leading inflammation and joint damage on RA. Cytokines regulate the phenotype of effector and regulatory T-cells in the synovium. Thus, an imbalance of cytokine network contributes to the development and progression of this autoimmune disease. In particular, TNF-α, IL-6, IL-1β and IFN-γ are considered to be disease promoting cytokines in RA [33,34]. In synovium, patients with RA had high frequencies of Th1 cells and of Th17 cells, which are thought to play a prominent pathogenic role in autoimmune arthritis. More recently, in plasma from RA patients, levels of IL-17, IL-23 and IFN-γ were significantly increased and correlated with a redox imbalance and oxidative damage [34]. Moreover, COMP, a specific serological marker which evaluates the articular cartilage degradation and its turnover, exerts an active role in inflammation. Increased plasma levels of COMP have been detected in the degenerating cartilage, synovial fluid and serum of patients with knee injuries and primary osteoarthritis (OA) and RA [35,36]. Among MMPs, MMP-3 has been reported to be the major enzymes produced by fibroblasts and macrophage cells in the synovium, and is responsible for the degradation of proteoglycan, various type of collagens and denatured type I and type II collagens, among others, besides its direct enzyme activity its activation is necessary for full activation of collagenases [17]. Our results revealed that those arthritic mice fed with Per-OL and OL-enriched-diets showed a significant reduction in serum MMP-3 and COMP levels as well as in IL-17, TNF-α, IL1-β, IFN-γ and IL-6 pro-inflammatory cytokines levels in paw tissues in comparison with SD-CIA control group which was correlated with the macroscopic and histological findings. These results are also in agreement with Castejón et at., who reported that OL controlled the production of inflammatory mediators decreasing IL-6 and TNF-α cytokines, MMP-1 and MMP-3 levels and mPGES-1 and COX-2 overexpression in human synovial fibroblast cell line SW982 [17]. 

The overexpression and activation of pro-inflammatory enzymes, including iNOS and COX-2, are known to intensify the disease severity of RA [37], which promoting inflammation and oxidative damage of the arthritic joint [38]. In fact, COX-2 and iNOS protein expressions are increased on rats CIA model and consequently, contributing to the progression of RA [39]. In accordance with Ryu et al. [16] who reported that OL suppressed the release of LPS-induced NO production and iNOS/COX-2 overexpression in RAW 264.7 murine macrophages, we have demonstrated that dietary Per-OL and OL treatments reduced COX-2 immunohistochemical expression in knee joints from DBA/1 mice CIA model and this was accompanied by a significant decrease on iNOS protein expression in paw homogenates from Per-OL fed mice reducing efficiently the joint damage. Therefore, regulation of these pro-inflammatory biomarkers by Per-OL could represent a potential molecular target susceptible to Per-OL modulation, which has not been demonstrated previously. 

Similarly, the main intracellular signal transduction pathways involved in RA include MAPKs, NF-κB and JAK/STAT pathways. Understanding of the signal transduction pathways implicated in RA has led to drug development programs targeting MAPK and NF-κB inhibitors [40,41]. In addition, a wide range of evidence indicates that Nrf2 transcription factor, may control different mechanisms involved in the physiopathology of rheumatic condition playing a central role in the protection of cells against oxidative stresses, up-regulating several genes en-coding anti-inflammatory and antioxidant proteins, such as HO-1 [42]. HO-1 gene expression activation is considered to be an adaptive cellular response to survive exposure to environmental stresses [26,43]. Moreover, some studies have revealed an interaction between members of the Nrf2 and NF-κB pathways, since that oxidative stress-induced NF-κB activation plays a pivotal role in the pro-inflammatory overproduction factors associated with the pathogenesis of RA and the activation of the Nrf2/ARE system disrupts this cycle [44]. In particular, Nrf2 inhibition aggravates cartilage destruction and accelerates the effector phase of RA in mice. NF-κB is considered a key signaling pathway in the control of synovial inflammation, hyperplasia and matrix generation [45] and is able to modulate the expression of several pro-inflammatory genes such as IL-1β, TNF-α, IL-6 and IL-17 in CIA experimental model [23]. High levels of these pro-inflammatory cytokines induce the recruitment of co-stimulary molecules which leading to the activation of NF-κB and MAPKs signaling pathways [25] which is responsible for COX-2 up-regulation [46]. For this reason, intra-articular or systemic blockade of NF-κB signaling pathway is an effective target in the management of RA [47]. Our data revealed that Nrf2 and HO-1 protein expressions decreased in SD-CIA control group; however, dietary OL and Per-OL treatments could restore Nrf2 and HO-1 expressions conferring a remarkable role of Nrf2/HO-1 signaling pathway in the beneficial effects of OL and Per-OL-enriched-diets in CIA model of RA. These results agree with Castejon et al., showed that both dietary OL and Per-OL treatments induced an activation of Nrf2/HO pathway and prevented the MAPK activation and nuclear NF-κB-p65 and NF-κB-p50 translocations by blocking IκB-α degradation in a pristane-induced SLE murine model. This was accompanied by a decrease on many pro-inflammatory markers production involved in this immunoinflammatory disease [21]. According to our results, induction of HO-1 expression may protect against cartilage destruction and decrease the secretion of pro-inflammatory cytokines in CIA model [48]. Moreover, up-regulating the expression of Nrf2 may exert anti-inflammatory effects in RA [49]. In this sense, our data suggest that Nrf2 overexpression observed in those arthritic animals fed with OL and Per-OL dietary treatments significantly could prevent the nuclear NF-κB transcription factor, resulting in an ameliorated pro-inflammatory markers production reducing the joint inflammatory injury. 

At the same time, MAPKs can participate in the regulation of NF-kappa B transcriptional activity. Furthermore, we determined that dietary OL and Per-OL treatments influenced the activation of MAPKs. It is well-established that MAPKs play important roles in transducing synovial inflammation and joint destruction and they are considered critical molecular targets for therapeutic intervention in RA [50]. JNK and ERK_1/2_ MAPKs are closely associated with collagenase production and inflammatory responses of fibroblast-like synoviocytes (RAFLS), whereas p38 MAPK isoforms is involved in regulating many of cellular biological processes, concretely synovial inflammatory cytokine production, which participate to the RA pathogenesis [45]. ERK_1/2_ also participates in promoting pannus formation and bone destruction in arthritic joints [51]. In the present study, the phosphorylation of all three kinases (ERK, JNK and p38 MAPK) was up-regulated by inflammation and was in accordance with the previous results obtained in pro-inflammatory cytokines profile determinations. On the contrary, OL and Per-OL experimental diets were able to prevent the phosphorylation of JNK, p38, and ERK1/2, interfering negatively with MAPK and NF-kappa B signaling pathways. 

## 5. Conclusions

In summary, our study showed, for the first time, the beneficial effects of dietary Per-OL and OL supplementation in CIA model of RA by reducing arthritic damage, serum pro-inflammatory biomarkers (MMP-3 and COMP), cytokines production (IL-6, IL-1β, TNF-α, IFN-γ, IL-17), and inhibiting both iNOS and COX-2 overexpression. The possible action mechanisms implicated in these beneficial effects could be related to an activation of the antioxidant Nrf2/HO-1 pathway as well as a blockage of MAPKs and NF-κB signaling pathways. Our results provide evidence for better anti-arthritic properties of Per-OL in comparison with OL and corroborate their potential as a new promising dietary strategy for the prevention and management of RA.
